# A new species of *Hyalella* (Crustacea, Amphipoda, Hyalellidae) from the Puna biogeographic province in Argentina

**DOI:** 10.3897/zookeys.865.32878

**Published:** 2019-07-22

**Authors:** Marcela Alejandra Peralta, Águeda Verónica Isa Miranda

**Affiliations:** 1 Instituto de Invertebrados, Fund. M. Lillo. Miguel Lillo 251, T4000JFE-San Miguel de Tucumán, Argentina Instituto de Invertebrados San Miguel de Tucumán Argentina

**Keywords:** Distribution, epigean, freshwater macroinvertebrates, South America, taxonomy

## Abstract

A new species of freshwater amphipod belonging to genus *Hyalella* is described from a peatbog at high altitudes (3,650 to 4,400 m above sea level) in the Puna region (Salta province, northwestern Argentina). The new species can be distinguished from other hyalellid species by the following combination of characters: dorso-posterior flanges on pleonites I–III; palp on maxilla 1 reaching almost half of distance between base of palp and base of setae on outer plate, and two papposerrate setae on the inner margin of inner plate of maxilla 2; propodus of gnathopod 1 hammer-shaped, inner face with seven serrate setae; propodus of gnathopod 2 ovate; male uropod 1 without curved seta on inner side of inner ramus; ramus of uropod 3 shorter than peduncle; six pair of sternal gills on pereionites II–VII.

A detailed morphological description and illustrations of the new species are provided. In Argentina, the new species represents the third record for the genus at altitudes greater than 2,000 m a.s.l., after *H.kochi* and *H.fossamancinii* (Dos Santos et al. 2008, González 2003), and the first record above 4,000 m a.s.l. Some comments about distributional and ecological aspects of the new species are included. With this new species, the number of *Hyalella* species known from Argentina and Falkland Islands (Islas Malvinas) rises to 12.

## Introduction

Within Amphipoda, the talitroid genus *Hyalella* Smith, 1874 is dominant in the surface freshwaters of South America. This genus is only known within the Neotropical and Nearctic regions. The natural environments inhabited by *Hyalella* include both surface (epigean) and groundwater (hypogean) habitats in a wide range of geographical heights, from sea level to more than 4,000 meters above sea level (a.s.l.).

At present, 73 species of *Hyalella* are known (Bastos-Pereira et al. 2018, Drumm and Knight-Gray 2019), but many remain undescribed. The highest diversity corresponds to Brazil, with 25 species (Streck et al. 2017). The species recorded so far in the freshwater environments of Argentina and the Falkland Islands (Islas Malvinas) are *H.curvispina* Shoemaker, 1942; *H.fossamancinii* Cavalieri, 1959; *H.pampeana* Cavalieri, 1968; *H.neonoma* Stock & Platvoet, 1991; *H.falklandensis* Bousfield, 1996; *H.rionegrina* Grosso & Peralta, 1999; *H.patagonica* Cunningham, 1871; *H.kochi* González & Watling, 2001; *H.bonariensis* Bond-Buckup, Araujo & Santos, 2008; *H.misionensis* Colla & César, 2015; and *H.pseudoazteca* González & Watling, 2003.

The Puna, a region that spans part of northeastern Chile, northwestern Argentina, southeastern Peru, and midwestern Bolivia, is characterized by the presence of endorheic basins at high elevations. In particular, the Puna peatbogs are freshwater bodies that function as natural sponges that hold and slowly release water, and thus help to regulate its transfer to surrounding areas. These peatbogs are ecosystems that hold the greatest biodiversity of the region and are highly vulnerable due to their ecological fragility and climate change (Vuille et al. 2008), which underscores the importance of furthering the knowledge of their biological diversity.

*Hyalella* is the most conspicuous taxon in the benthic macroinvertebrate communities of the Puna Mountains in Salta province, Argentina (Nieto et al. 2017). As part of the results of a project for the study of freshwater macroinvertebrates (Fundación Miguel Lillo), a new *Hyalella* species is described herein. The new species occurs in the Puna biogeographic region, in wetlands at altitudes greater than 4,000 m a.s.l. in the Argentinian Andes.

## Materials and methods

Samples were collected with the aid of a net and fixed in formaldehyde or 96% ethanol in situ. Once in the laboratory, specimens were transferred to 80% ethanol and dissected in Brunson solutions (glycerin, ethanol, and distilled water) under a stereomicroscope, and appendages illustrated using a Leitz Dialux camera lucida. Body measurements were made from the tip of the head to the tip of the telson. Photographs of the male paratype were taken with the aid of a stereomicroscope LEICA M165 C coupled with LEICA DMC 2900 digital camera. The geographic distribution map was digitally generated using the shapefiles from Arana et al. (2017) with the Esri ArcGIS 10.5 desktop software.

The terminology for setae follows Zimmer et al. (2009). The description of the new species was made based on previous taxonomic works on *Hyalella* species (Grosso and Peralta 1999, González and Watling 2001, 2003, Bastos-Pereira and Bueno 2012). Specimens are deposited in the Crustaceans Collection of Fundación Miguel Lillo, Tucumán, Argentina (**FML**).

## Taxonomy

### Order Amphipoda Latreille, 1816

#### Family Hyalellidae Bulycheva, 1957

##### Genus *Hyalella* Smith, 1874

###### Hyalella puna sp. n.

Taxon classificationAnimaliaAmphipodaHyalellidae

89210024-a24c-45e6-8677-08c621518009

http://zoobank.org/1EEAB3A6-AF20-4FE2-925D-383AA5E09AC4

**Figure 1.**
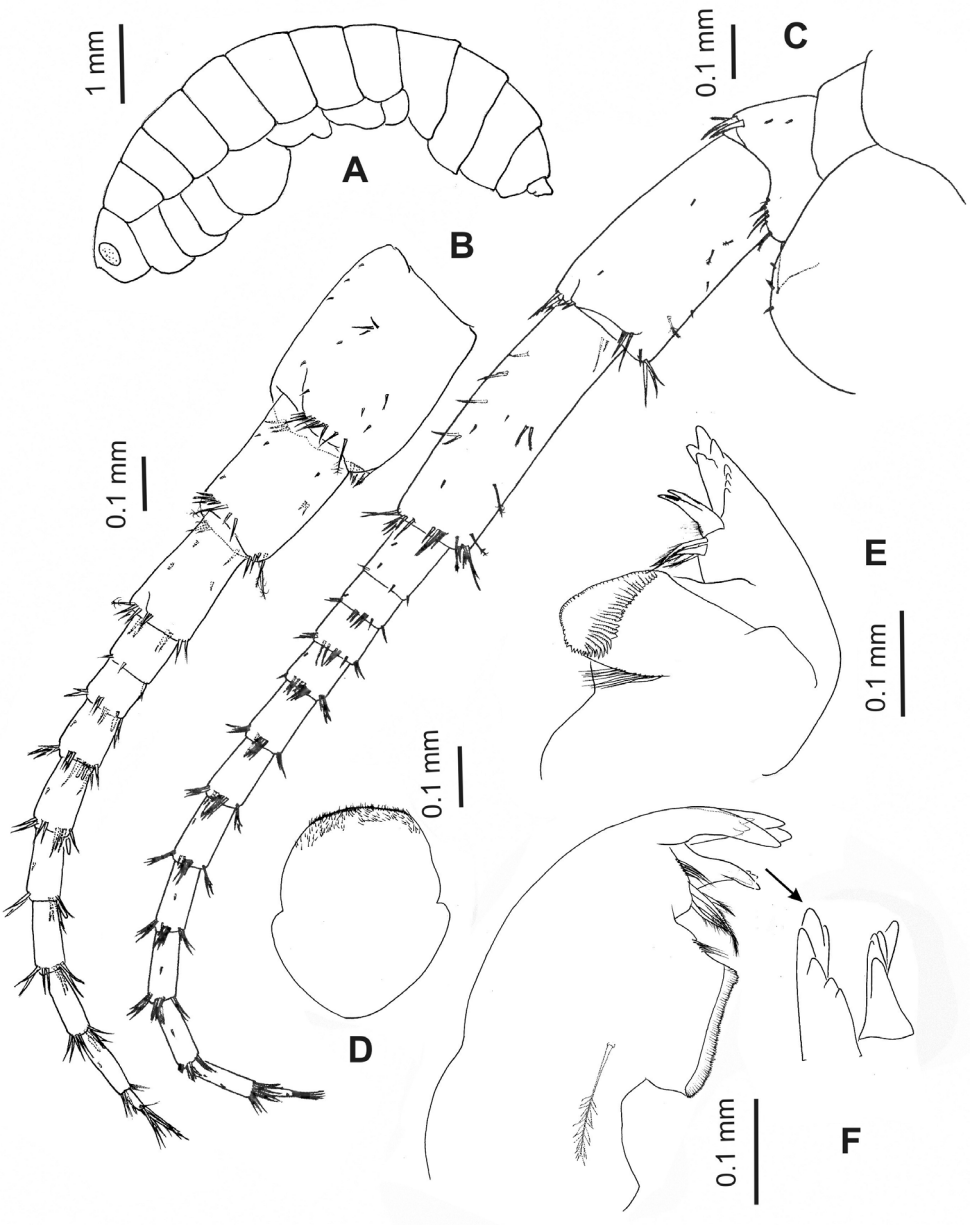
*Hyalellapuna* sp. n., male holotype. **A** lateral view of habitus **B** antenna 1 **C** antenna 2 **D** labrum **E** right mandible **F** left mandible, arrow indicates detail of incisor and lacinia.

**Figure 2.**
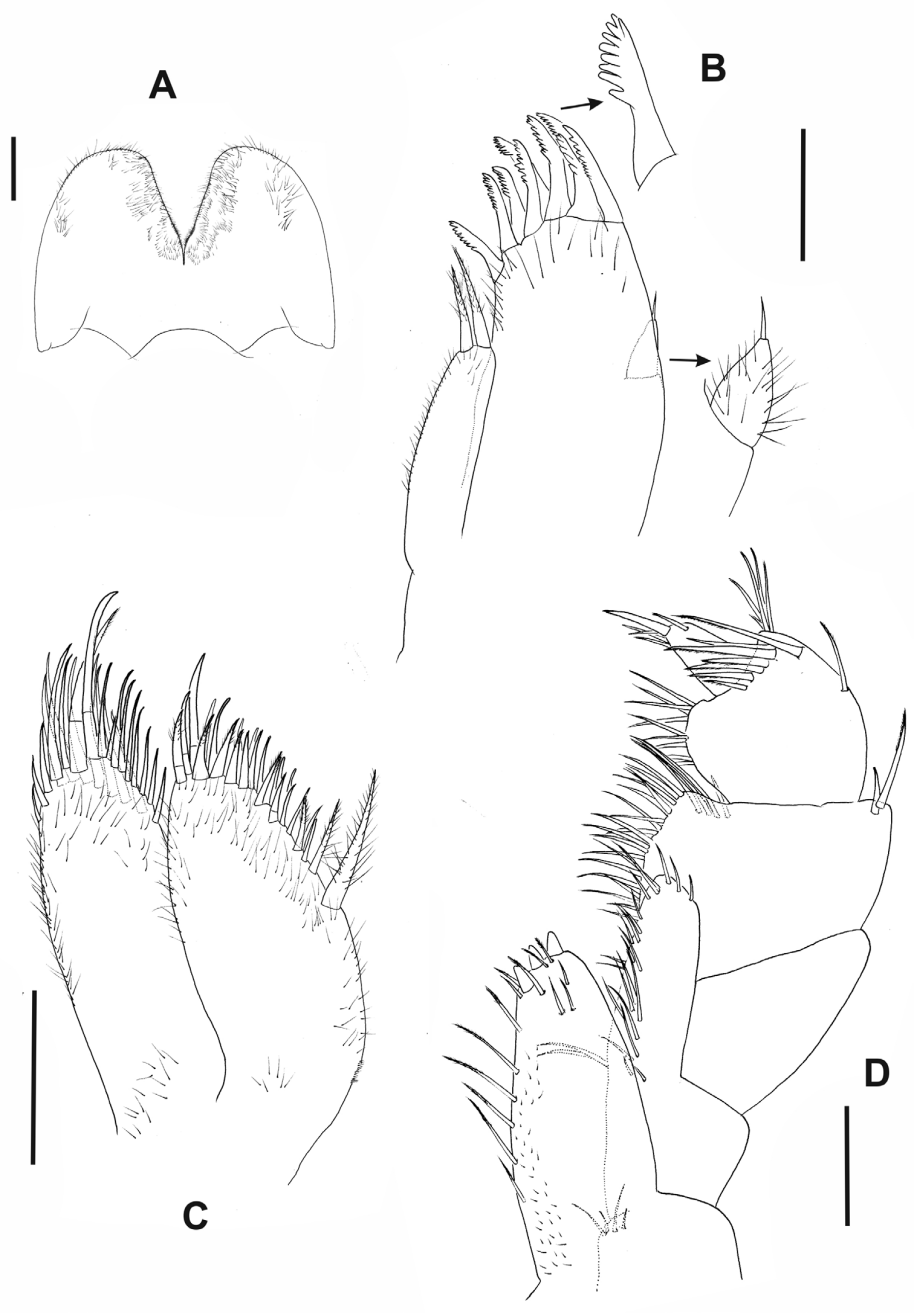
*Hyalellapuna* sp. n., male holotype. **A** lower lip **B** maxilla 1, arrows indicate details of seta from outer plate and palp **C** maxilla 2 **D** right maxilliped. Scale bars: 0.1mm.

**Figure 3.**
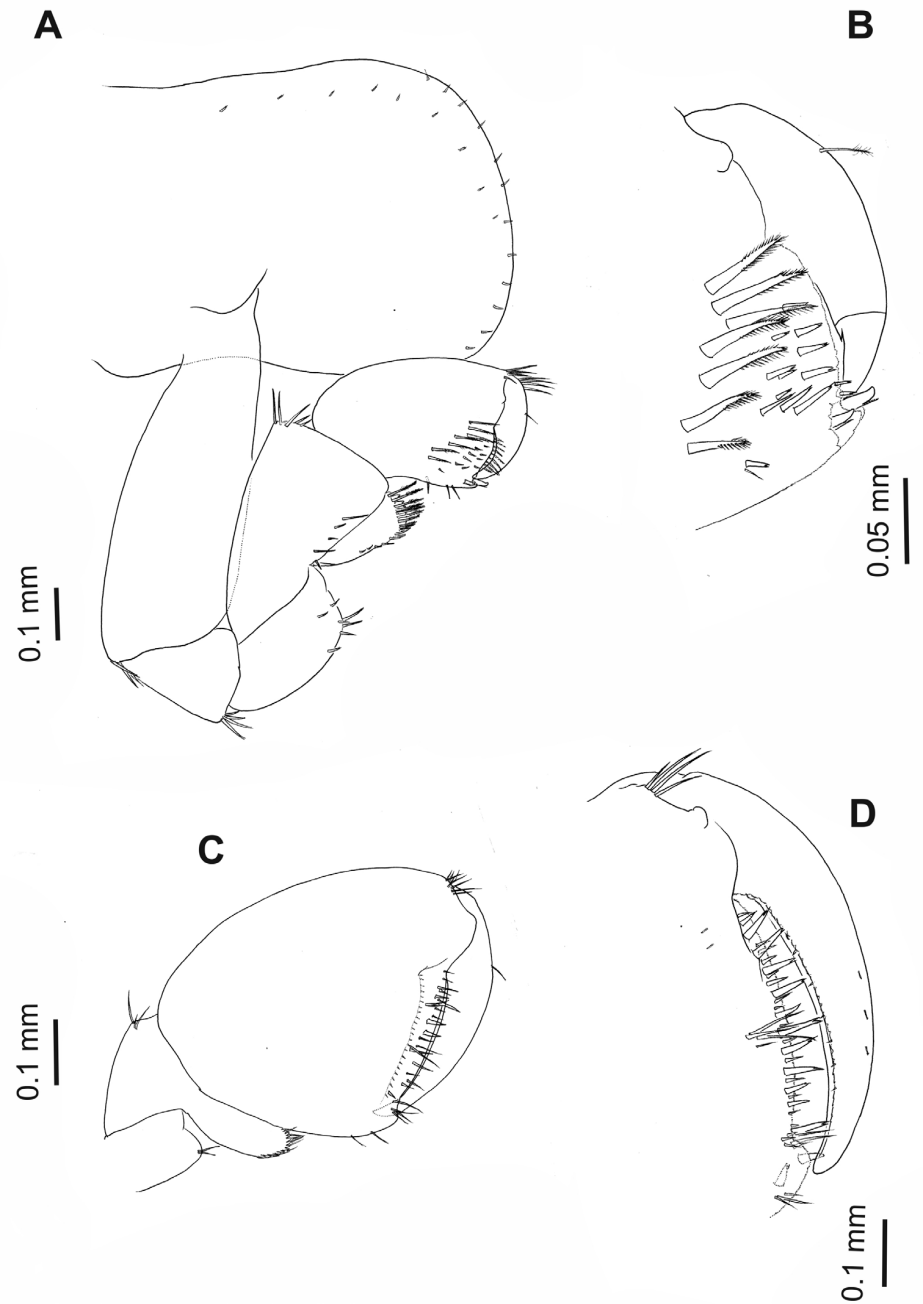
*Hyalellapuna* sp. n., male holotype. **A** gnathopod 1 **B** gnathopod 1, detail of propodus and dactylus **C** gnathopod 2 carpus, propodus and dactylus **D** gnathopod 2, detail of propodus, and dactylus.

**Figure 4.**
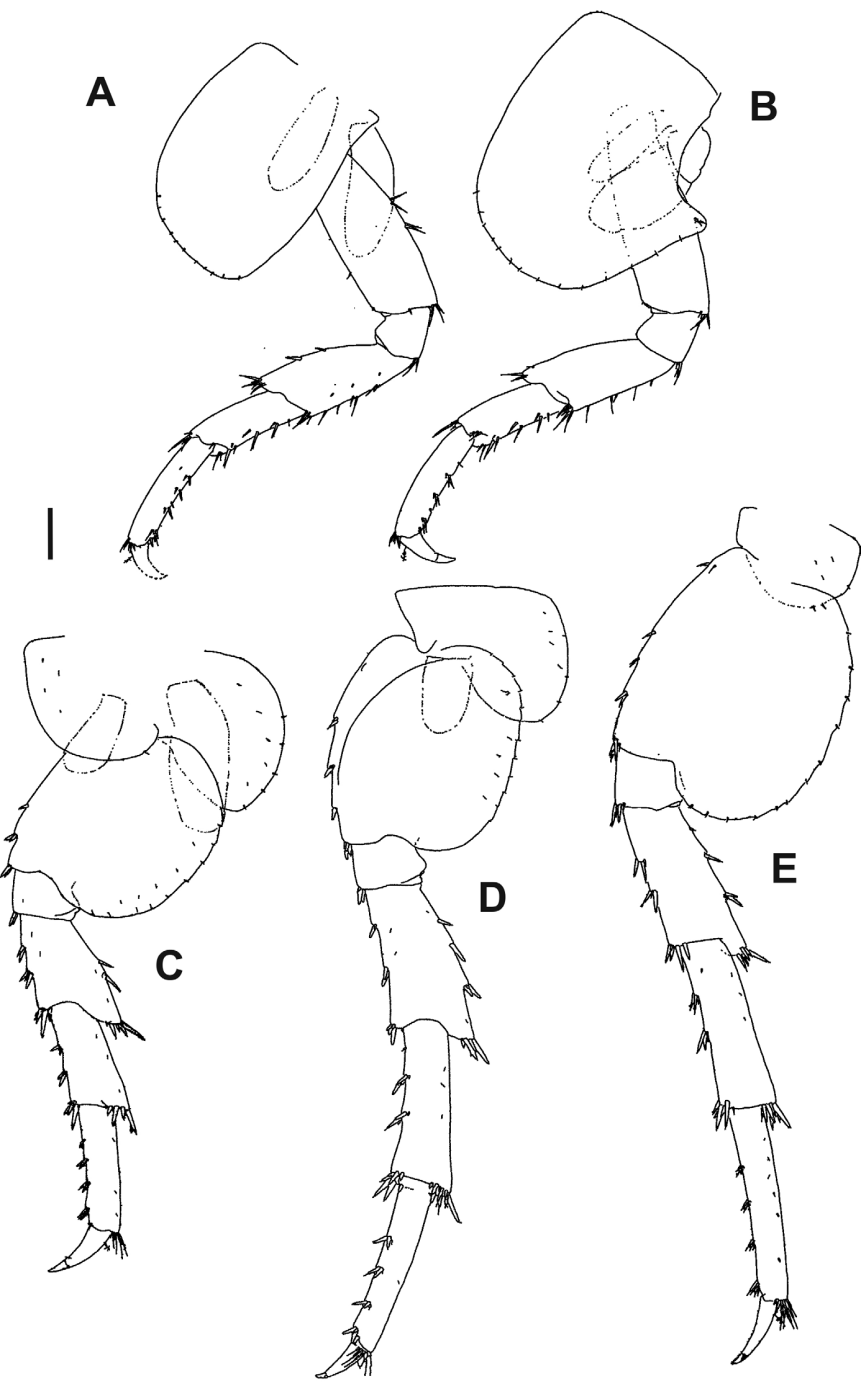
*Hyalellapuna* sp. n., male holotype. **A** pereiopod 3 **B** pereiopod 4 **C** pereiopod 5 **D** pereiopod 6 **E** pereiopod 7. Scale bar: 0.2 mm.

**Figure 5.**
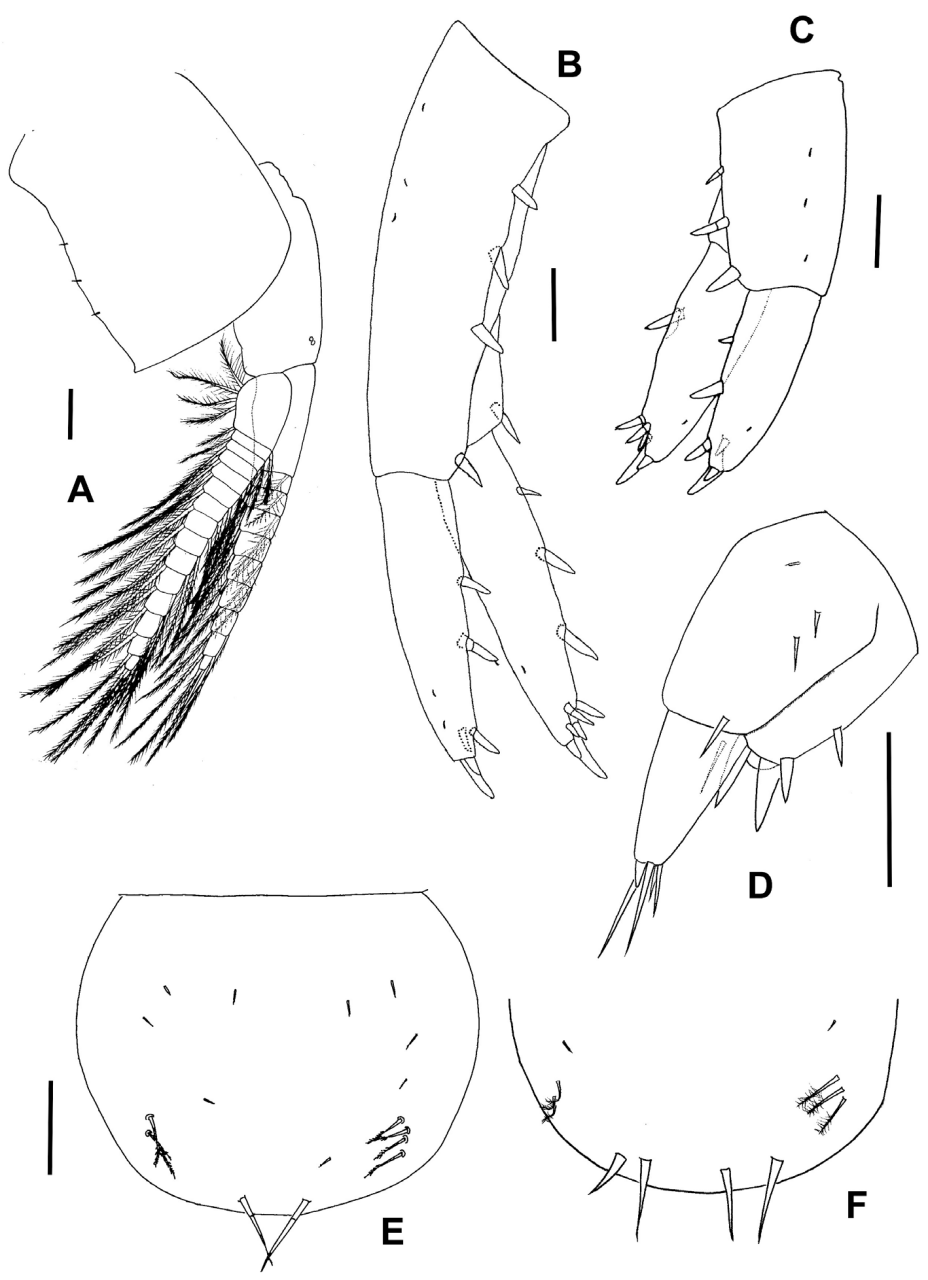
*Hyalellapuna* sp. n., male holotype. **A** pleopod 3 **B** uropod 1 **C** uropod 2 **D** uropod 3 **E** telson **F** telson of male paratype. Scale bars: 0.1mm.

**Figure 6.**
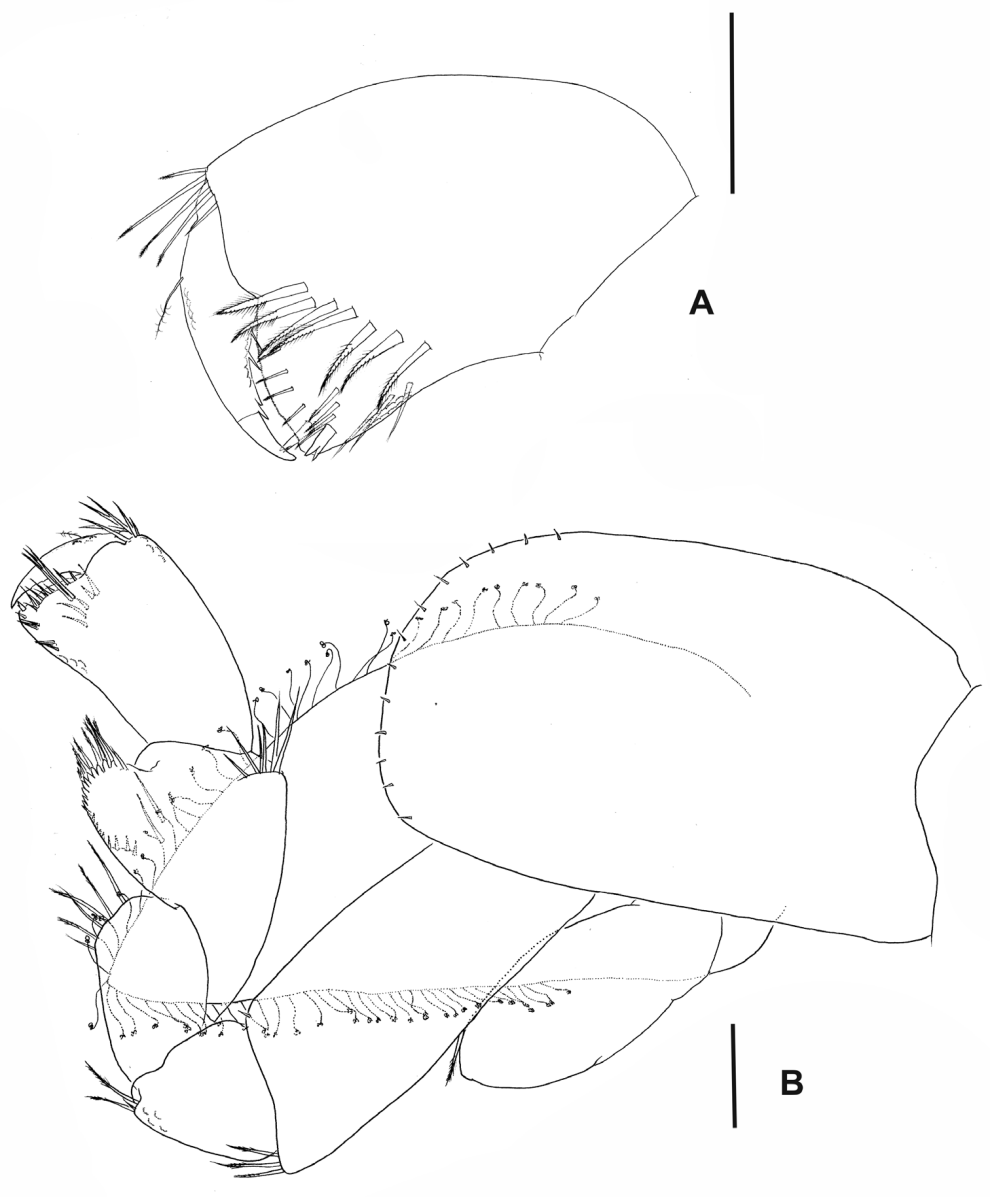
*Hyalellapuna* sp. n., female. **A** gnathopod 1, propodus and dactylus **B** gnathopod 2. Scale bars: 0.1mm.

**Figure 7.**
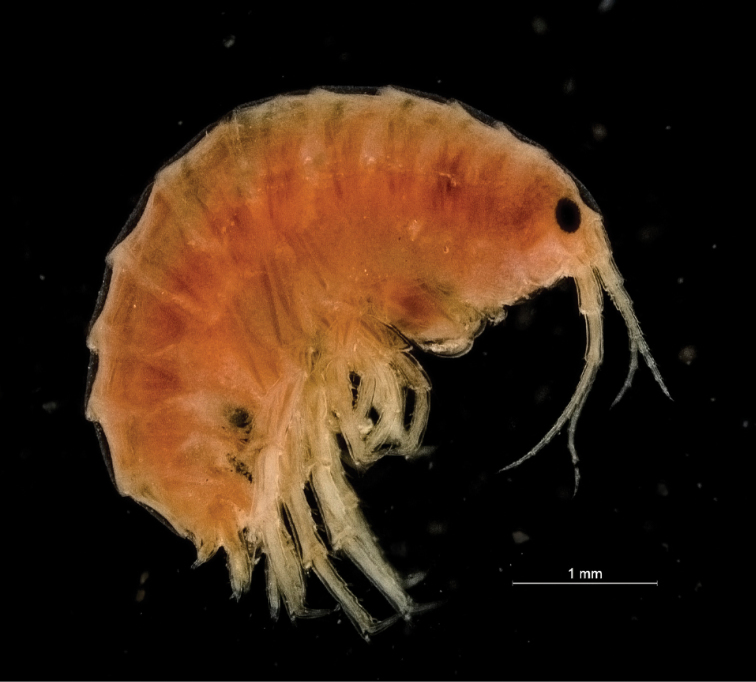
*Hyalellapuna* sp. n., male paratype, habitus.

Figs 1 , 2 , 3 , 4 , 5 , 6 , 7

####### Type material.

***Holotype***: Argentina: ♂, 7.42 mm; Salta, La Poma; 24°30'S, 66°47'W; 4,400 m a.s.l.; 11 Nov. 2000; C. Locascio de Mitrovich leg.; peatbog close to Santa Rosa de los Pastos Grandes, depth 5 cm (FML-CRUST 01261).

***Paratypes***: Argentina: ♀; same data as for holotype (FML-CRUST 01262). 9 ♂♂; same data as for holotype (FML-CRUST 01263). 6 ♀♀ same data as for holotype (FML-CRUST 01264).

####### Other material.

Argentina: 9 ♂♂, 9 ♀♀; Salta, Vega Los Patos; 25°23'S, 66°54'W; 4,120 m a.s.l; 25 Jan 2010; C. Locascio de Mitrovich leg. (FML-CRUST 01265). 9 ♂♂, 8 ♀♀; Jujuy, Cuenca Pozuelos, Pocitos; 22°27'S, 66°00'W; 3,650 m a.s.l.; 23 Jan 2010; A. González Aschem leg.; Physicochemical water parameters, temperature 14 °C, pH 7.5, 0.24 ms conductivity, 157 ppm total solids, 5.2 mg/l OD, sat.O2: 3.2, depth 20 cm (FML-CRUST 01200).

####### Type-locality.

Argentina, Salta, La Poma; 24°30'S, 66°47'W; 4,400 m a.s.l.; peatbog close to Santa Rosa de los Pastos Grandes, depth 5 cm; 11 Nov. 2000; C. Locascio de Mitrovich leg.

####### Diagnosis.

Body with dorso-posterior flanges on pleonites I–III. Eyes pigmented, ovoid. Antenna 1 shorter than antenna 2. Palp of maxilla 1 longer than wide, reaching almost half of distance between base of palp and base of setae on outer plate; inner plate slender, with two strong and papposerrate apical setae. Inner plate of maxilla 2 with two unequal strong papposerrate setae on inner margin. Propodus of gnathopod 1 length less than two times its maximum width, hammer-shaped, inner face with seven serrate setae; comb-scales on disto-posterior and disto-anterior border. Propodus of gnathopod 2 ovate, palm shorter than posterior margin, without notch, slope transverse, anterior edge smooth. Uropod 1 not sexually dimorphic. Peduncle of uropod 3 with three strong and two thin distal setae and other thin marginal setae; ramus shorter than peduncle. Six pair of sternal gills on pereionites II–VII.

####### Etymology.

Species name refers to the Puna biogeographic province.

####### Habitat.

Freshwater, epigean.

####### Accompanying fauna and algae.

Diptera (Chironomidae), Coleoptera (Staphylinidae and Elmidae); Heteroptera; Ephemeroptera; CrustaceaCopepoda (Cyclopoida and Harpacticoida); anuran tadpoles; algae *Nostoc*.

####### Description of Holotype male.

Size, 7.42 mm. Head smaller than first two thoracic segments. Eyes pigmented, large, ovoid. Body with dorso-posterior flanges on pleonites I–III. Epimeral plate I round, plates II and III acuminate. Coxae I to III subequal in size and shape, slightly overlapping. Acumination in coxae absent. Coxa III narrower than IV. Coxa IV as wide as deep, excavated posteriorly. Posterior lobe of coxa V deeper than anterior lobe. Anterior lobe of coxa VI small (Figs 1A, 7).

Antenna 1 (Fig. 1B) much shorter than antenna 2 but longer than peduncle of latter; peduncle longer than head, all segments of peduncle with group of plumose or simple setae and microtrichs, first segment ⅓ longer than second one, third segment slightly shorter than second. Flagellum of nine articles, with group of simple setae, 1–2 aesthetascs per article occurring distally between articles 2–7, distal article with group of eight long setae.

Antenna 2 (Fig. 1C) with peduncle longer than head, article 4 slightly shorter than article 5; articles 3–5 with distal groups of simple setae and microtrichs; article 4 and 5 with medial plumose simple setae. Flagellum with 12 articles, distally with group of five simple setae, each article with distal group of simple setae and medial microtrichs.

Labrum (Fig. 1D) ventral margin truncate, covered by short distal setules.

Mandibles basic amphipodan type (sensu Watling 1993); each with well-developed molar large, cylindrical, triturative. Left mandible (Fig. 1F) incisor 6-denticulate (three short, three long); lacinia 5-denticulate; setal row with four pappose setae. Right mandible (Fig. 1E) incisor 8-denticulate (four short, four long); lacinia complex, with multi-denticles, setal row with four pappose setae.

Lower lip (Fig. 2A) outer lobes rounded with distal, internal, and external setules, mandibular projection of outer lobes truncated.

Maxilla 1 (Fig. 2B) palp uniarticulated, reaching almost half of the distance between base of palp and base of setae on outer plate; nine serrate distal setae on outer plate; inner plate slender, shorter than outer plate, bearing two apical papposerrate setae.

Maxilla 2 (Fig. 2C) inner plate subequal in length to outer plate; inner plate with two unequal robust papposerrate setae proximally on inner margin; outer plate with several apical simple setae; outer and inner plates with several setules.

Maxilliped (Fig. 2D) inner plate apically rounded, longer than wide, with three cuspidate setae, apex and inner margins with pappose and simple setae; outer plate with apical and medial simple setae; palp longer than outer plate, with four articles; inner margins of articles 2 and 3 with long simple setae; outer distal face of article 3 with cluster of simple setae, distal margin with simple and serrated setae; article 4 unguiform, shorter than article 3, distal setae simple and shorter than nail.

Coxal gills (Fig. 4A–C) on gnathopod 2 to pereiopod 6, sac-like. Sternal gills tubular on pereionites II–VII (Fig. 4A–D).

Gnathopod 1 (Fig. 3A, B) subchelate; basis and ischium with cluster of setae on disto-posterior border; carpus longer than wide, with strong and wide concave posterior lobe, border pectinate and with several serrate setae; propodus length less than two times maximum width, hammer-shaped, without setae on anterior border, inner face with seven serrate setae, and small simple setae, comb-scales on disto-posterior and disto-anterior border, palm slope slightly transverse, margin convex, palm angle with two cuspidate setae with accessory seta; dactylus claw-like, congruent with palm. Palmar Index (sensu Ruffo 1973) = 0.34.

Gnathopod 2 (Fig. 3C, D) subchelate; posterior lobe of carpus elongated, border pectinate with several serrate setae; propodus ovate, comb-scales on disto-posterior margin, palm margin shorter than posterior margin, slope transverse, palm margin straight and regular with several strong short and medium-length setae, few long setae, anterior edge smooth, disto-anterior border with cluster of thin simple setae, palm angle with two cuspidate setae with an accessory seta; dactylus claw-like with several endal setae and comb-scales, congruent with palm, with one thin plumose seta dorsally. Palmar Index (sensu Ruffo 1973) = 0.48.

Pereiopods 3–4 (Fig. 4A, B) similar in size and shape; posterior margins of carpus and propodus with cuspidate and simple setae, posterior margin of merus with simple setae; dactylus less than half the length of propodus, with a plumose seta. Coxal plates: pereiopod 3: longer than wide; pereiopod 4: excavated posteriorly, as long as wide; all coxal plates with small simple setae on margins.

Pereiopods 5–7 (Fig. 4C–E) pereiopod 5 distinctly shorter than 6 and 7, the latter two subequal in length; posterior margin of basis of pereiopods 5–7 expanded (more so in 5 and 7 than in 6) and finely serrate; anterior margins of merus, carpus and propodus with ten marginal clusters of 2–6 cuspidate seta; dactylus less than half the length of propodus, with a plumose seta. Coxal plates: pereiopod 5: wider than long, with two lobes, posterior lobe longer; pereiopod 6: wider than long, with two lobes, anterior lobe much reduced; pereiopod 7: wider than long; all coxal plates with small simple setae on margins.

Pleopods (Fig. 5A) all similar, peduncle shorter than rami, biramous, rami multi-annulated and bearing long plumose setae.

Uropod 1 (Fig. 5B) peduncle longer than rami, with a longitudinal row of three cuspidate setae on dorsal surface and two additional distal setae; rami subequal in length; outer ramus with two dorsal and four distal cuspidate setae; inner ramus with three dorsal and six terminal setae; without curved seta on inner side of inner ramus.

Uropod 2 (Fig. 5C) shorter than uropod 1; peduncle with three setae in a longitudinal row; rami subequal; inner ramus with one dorsal cuspidate seta, apex with six cuspidate setae; outer ramus with two cuspidate setae, one of them shorter, distributed along the ramus, and apex with four cuspidate setae.

Uropod 3 (Fig. 5D) shorter than peduncle of uropod 1, as long as peduncle of uropod 2; peduncle quadrate, wider than ramus, with three strong and two thin distal setae and other thin marginal setae; outer ramus uniarticulated, shorter than peduncle, basal width 1.5 times or less tip of ramus, with four simple distal setae, one very short cuspidate seta with accessory seta.

Telson (Fig. 5E) wider than long, entire, apically rounded, bearing two long simple setae symmetrically distributed on distal margin, and three or four small plumose setae close to each main seta, occasionally with four long simple setae on apical margin (Fig. 5F).

####### Characters of female that differ from male.

Female mean body size: 7.78 mm (7 individuals). Presence of foliaceous oostegites, with curl-tipped setae on the margin, on pereionites II–V (Fig. 6B). Both pairs of gnathopods in females similar in size and shape, inner face of propodus of gnathopod 1 with seven serrate setae (Fig. 6A). Gnathopod 2 (Fig. 6B) different from male gnathopod 2 in shape and smaller, propodus subrectangular, 2–3 times as long as its maximum width, with row of five serrate setae on inner face, palm transverse, subchelate.

####### Variability.

Measurements: Body length ranged from 7.42 to 8.80 mm (males) and 7.33 to 8.13 mm (females). Male mean body size: 7.89 mm (10 individuals). Female mean body size: 7.78 mm (7 individuals). The number of articles of flagellum in antenna 1 varied from 7 to 10 (males) and 7 to 11 (females). In antenna 2 this number ranged from 9 to 14 (males) and 8 to 13 (females).

####### Taxonomic remarks.

This new species can be distinguished from the other Argentine species of *Hyalella* by the flanges on pleonites I, II and III. Beyond this, *Hyalellapuna* sp. n. is similar to *H.kochi* in its general morphology, but bearing six sternal gills from pereionites II to VII (*H.kochi*: five sternal gills on pereionites III–VII); ramus of uropod 3 shorter than its peduncle (*H.kochi*: ramus and peduncle equal in length); male uropod 1 without curved seta on inner ramus (*H.kochi*: with curved seta); palp of maxilla 1 longer than wide, reaching almost half of the distance between base of palp and base of setae on outer plate (*H.kochi*: shorter palp); and inner plate of maxilla 2 with two strong pappose setae on inner margin (*H.kochi*: inner plate of maxilla 2 with only one pappose seta).

**Figure 8.**
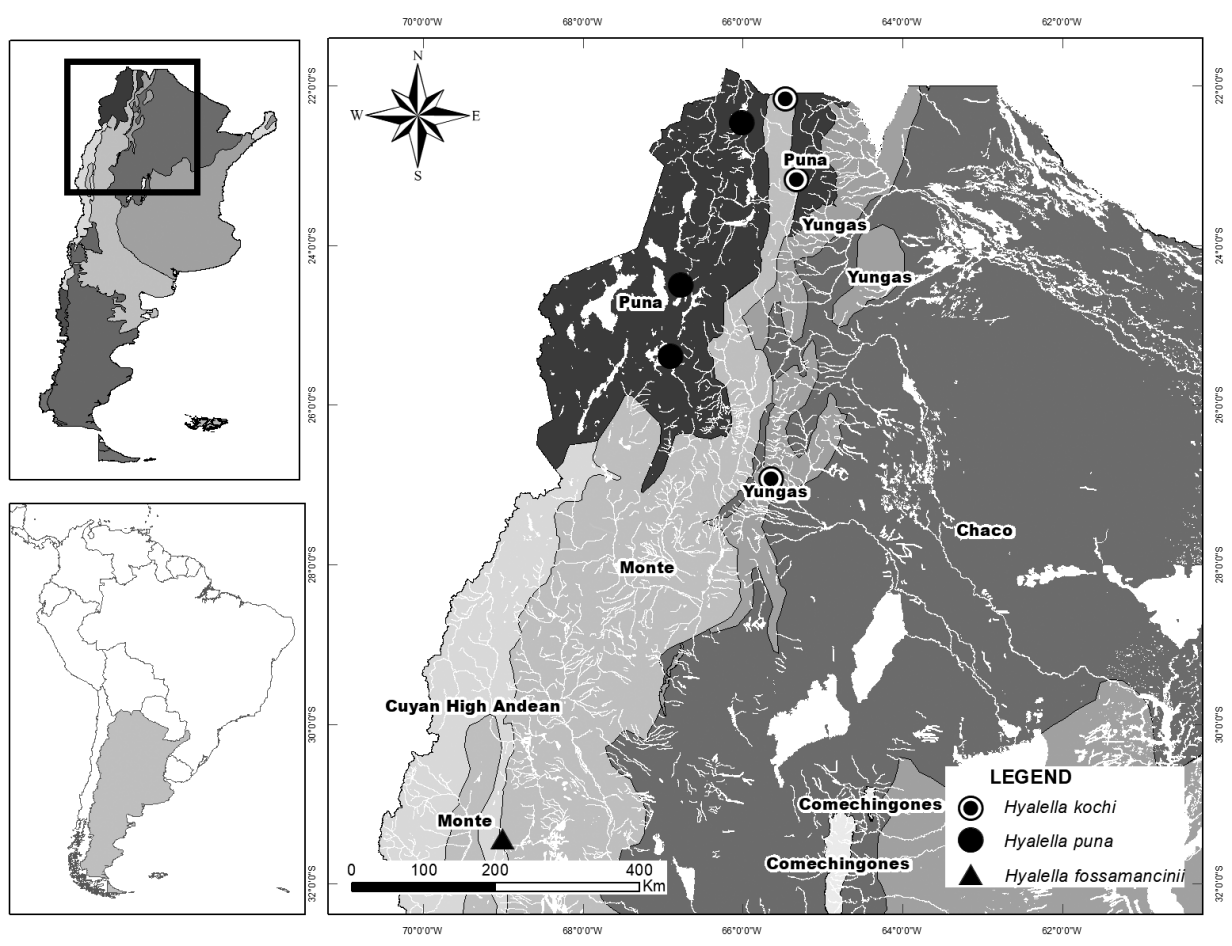
Distribution map of *Hyalellakochi*, *H.fossamancinii*, and *H.puna* sp. n. in northwestern Argentina within a biogeographic scheme.

Table 1 presents a comparison of the main morphological characters of *H.puna* sp. n. and the other Argentinian *Hyalella* species from high elevations, namely *H.kochi* and *H.fossamancinii* (see Fig. 8 for geographical distribution of each species).

**Table 1.** Male characters of high-altitude *Hyalella* species from Argentina. Key: A1: Antenna 1; A2: Antenna 2; G1: Gnathopod 1; G2: Gnathopod 2; U1: Uropod 1; U3: Uropod 3.

Characters	*H.puna* sp. n.	*H.kochi* González & Watling, 2001	*H.fossamancinii* Cavalieri, 1959
A1: articles of flagellum	9–10	9	9–10
A2: articles of flagellum	10–14	11	9–14
Body length (mm)	7.79 (mean)	6.9	9.42
G1: comb-scales on propodus	present	present	absent
G1: setae on inner face	7	7	more than 10
U1: curved seta on inner ramus	absent	present	absent
Sternal gills on pereionites	II–VII	III–VII	III–VII
Telson: simple apical setae	2–4 long	2 long	12 short
Dorsoposterior flanges on pleonites I–III	present	absent	absent
Maxilla 1: palp length	reaching almost half of the distance between base of palp and base of setae on outer plate	reaching less than a third of the distance between base of palp and base of setae on outer plate	reaching more than half the distance between base of palp and base of setae on outer plate
Maxilla 2: strong paposerrate setae on inner plate	2	1	1
U3: proportion between length of ramus and peduncle	outer ramus shorter than peduncle	subequal	outer ramus shorter than peduncle
Distribution	Salta province: La Poma department, peatbog close to Santa Rosa de los Pastos Grandes (4,400 m). Jujuy province: Cuenca Pozuelos, Pocitos (3,650 m).	Jujuy province: La Quiaca, Yavi Chico River (3,432 m); reservoir near Escuela Agrotécnica in Humahuaca, (2,998 m); Tilcara, a lake north from Tilcara, near Rio Grande, (2,503 m). Tucumán province: Los Sosa River on Route 307 in the direction of Tucumán with Tafí del Valle (1,855m)	San Juan province: Bramadero River, Santa Cruz River (3,500 m), Las Arenas lagoon, Valle Hermoso River, Patillos River.

####### Ecological and distribution remarks.

The type locality of *Hyalellapuna* sp. n. is within the Pastos Grandes sub-basin, an area with 90–100 mm total annual rainfall (Nieto et al. 2016). This sub-basin belongs to the “Cuenca Cerrada de la Puna” basin (Nieto et al. 2016). The benthic fauna of the Puna wetlands in Argentina and Chile consists mostly of undetermined *Hyalella* species (Scott et al. 2015, Rodrigues Capítulo et al. 2014, Nieto et al. 2016). In these high Andean wetlands, the macroinvertebrate communities have varying tolerances to different ranges of salinities. Electric conductivity was the second main characteristic associated to compositional changes of the benthic macroinvertebrate communities (Nieto et al. 2017); thus, decreases in the abundance, taxonomic richness, and diversity of various zoobenthic taxa have been attributed to an increase of salinity and conductivity (Rodrigues Capítulo et al. 2014). However, the data reported for physicochemical parameters here (Pozuelos basin, FML-CRUST 01200) and in previous studies of Puna wetlands from northwestern Argentina (Scott et al. 2015; Rodrigues Capítulo et al. 2014; Nieto et al. 2016) have shown that *Hyalella* species are generally more tolerant to greater salinity and conductivity levels (191.15 to 2,203 μS/cm approx.) than other taxa.

Little is known about the distribution of benthic macroinvertebrates at high altitudes of the Andean region. *Hyalellapuna* sp. n. is known only from high altitude areas, and occurs mostly within the biogeographic Puna province. In Argentina, the new species represents the third record for the genus at altitudes greater than 2,000 m a.s.l., after *H.kochi* and *H.fossamancinii* (Dos Santos et al. 2008, González 2003), and the first record above 4,000 m a.s.l.

If the distribution of the new species is included in a previous panbiogeographic analysis of *Hyalella* species (De Los Ríos Escalante et al. 2012), it is consistent with the ‘Central Andes’generalized track. The latter includes inland waters from central Argentina (31°S) to northern Chile (18–26°S). The species assigned to this track are *H.fossamancinii* and *H.kochi* (De Los Ríos Escalante et al. 2012).

## Supplementary Material

XML Treatment for Hyalella puna
